# Supplementary material for: Adaptive coding across visual features during free-viewing and fixation conditions
Source: Nat Commun. 2023 Jan 6;14:87. doi: 10.1038/s41467-022-35656-w (PMC9816177; doi:10.1038/s41467-022-35656-w)
Supplement: Supplementary file 1 — Supplementary Information [file 41467_2022_35656_MOESM1_ESM.pdf]

Supplementary Information

Adaptive coding across visual features during free-viewing and fixation conditions

Sunny Nigam, Russell Milton, Sorin Pojoga, Valentin Dragoi

R	G	B	u	v	L (cd/m^2)
173	0	1	0.273	0.548	9.32
143	71	0	0.204	0.556	9.29
117	86	0	0.155	0.551	9.36
81	99	0	0.109	0.566	9.46
0	104	67	0.093	0.516	9.33
0	100	83	0.105	0.473	9.30
0	97	88	0.107	0.455	9.43
0	96	91	0.108	0.446	9.30
0	96	96	0.112	0.434	9.47
0	95	101	0.115	0.420	9.48
0	95	117	0.126	0.382	9.33
118	0	181	0.189	0.236	9.36
152	0	129	0.219	0.341	9.30
159	0	100	0.230	0.393	9.32
164	0	94	0.241	0.430	9.34
168	0	71	0.255	0.483	9.33
Neutral gray stimulus					
88	88	88	0.137	0.451	9.35

Supplementary Table 1. Color stimulus properties. Luv coordinates and luminance values for color stimuli and neutral gray screen. Mean luminance 9.35 ± 0.01 cd/m^2.

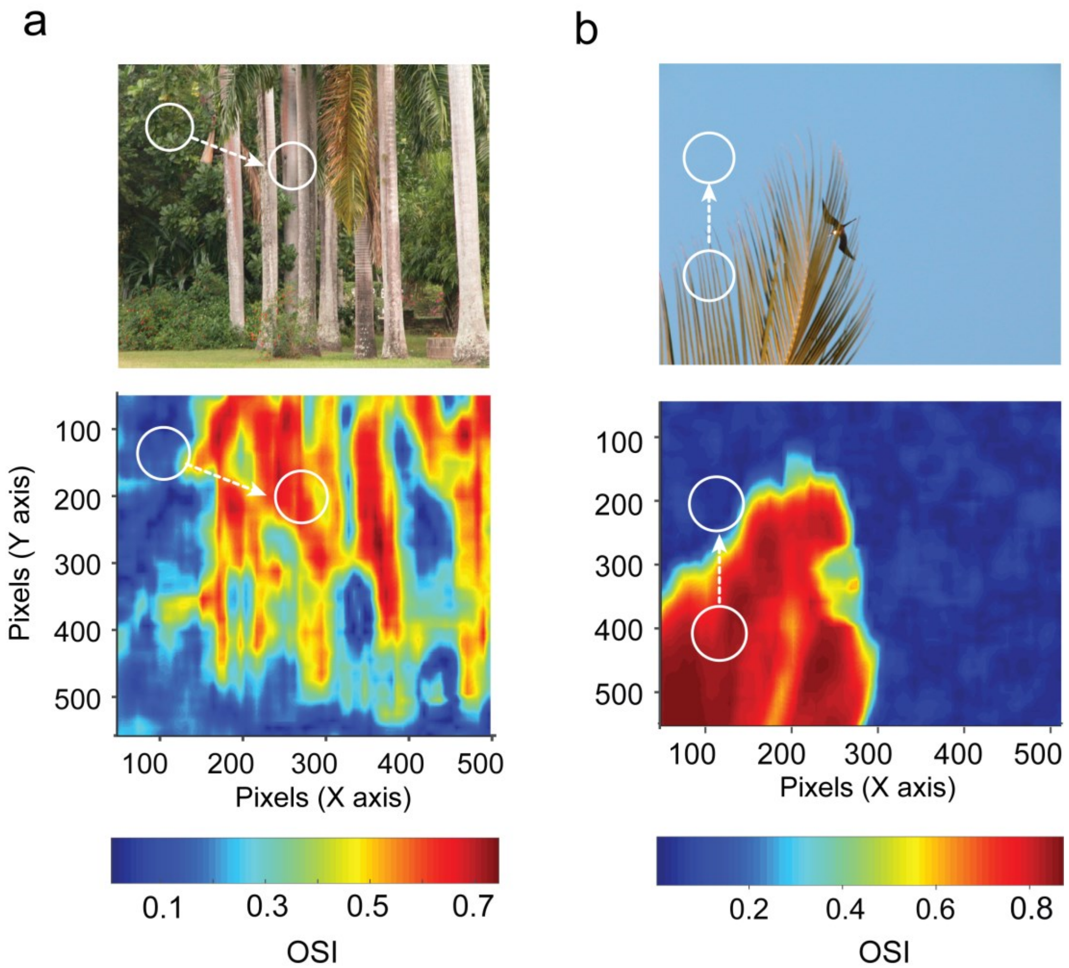

Supplementary Fig. 1 Local orientation analysis of natural scenes. (a) Natural scene (top, McGill calibrated color image database[11]; Olmos, A., Kingdom, F. A. A. (2004). A biologically inspired algorithm for the recovery of shading and reflectance images, Perception, 33, 1463 - 1473. http://tabby.vision.mcgill.ca/) and pixel by pixel characterization of mean orientation quantified by Orientation Selectivity Index (OSI) using a Sobel filter. **(b)** Same as in **a** except for a different example natural scene[11]. White circles represent schematic description of position of receptive fields and how feature properties change due to eye movements (dotted white arrow) from one fixation to the other.

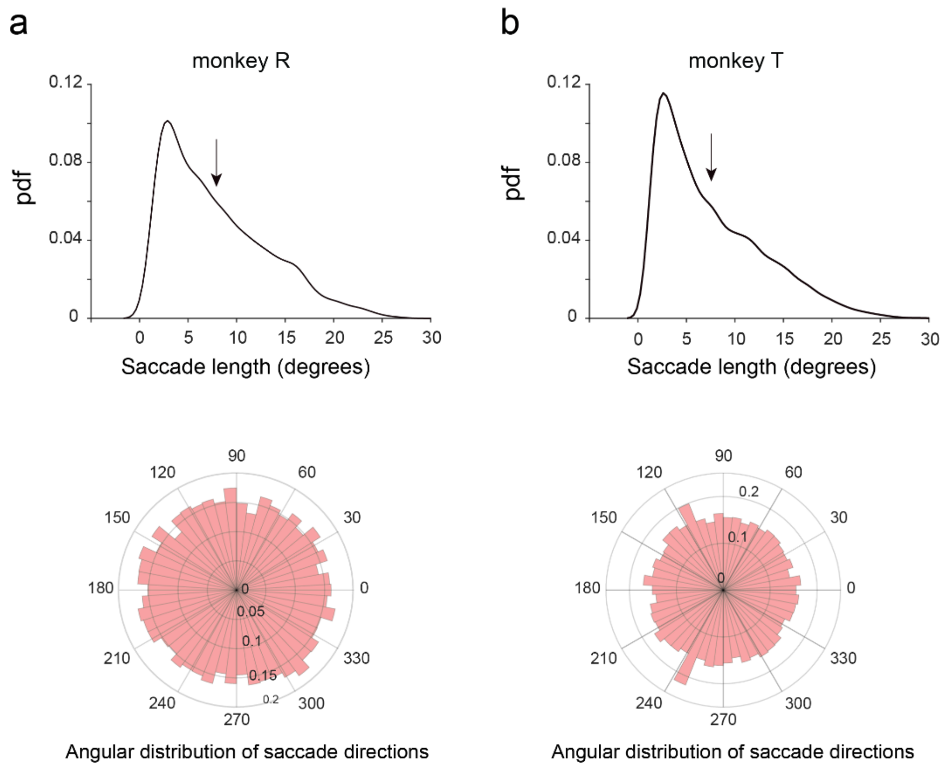

Supplementary Fig. 2 Distribution of saccade length and direction during free-viewing. (a) Top: Probability density function of saccade lengths (n = 11253, mean = 7.9 ± 0.05 deg) made by monkey R, pooled across all sessions during free-viewing of stimuli described in Fig. 1e. Bottom: Distribution of the direction of saccades (circular mean = 1.2°) for the same animal. (b) Top and bottom represent the saccade length and direction distributions for monkey T (n = 25250, mean saccade length = 7.6 ± 0.03 deg and circular mean = -2.8°).

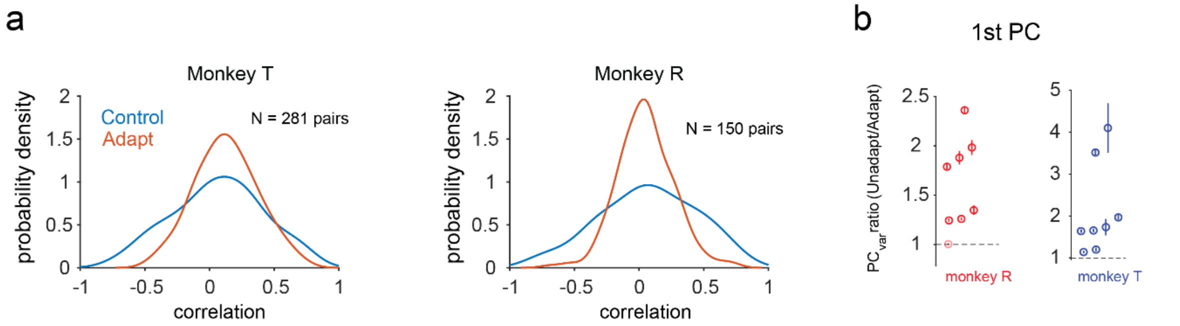

Supplementary Fig. 3 Cross feature adaptation modifies the structure of pairwise and population correlations in free-viewing experiments. (a) Probability density function of mean noise correlations (N = 281 pairs, monkey T; N = 150 pairs, monkey R) averaged over test stimuli in adapted (orange) and unadapted (blue) conditions. (Monkey T: σ^2_{adapt} =0.059, $\sigma^2_{unadapt}$ =0.120; two-sample F-test, P = 1.9e-7, Monkey R: σ^2_{adapt} =0.046, $\sigma^2_{unadapt}$ =0.140; two-sample F-test, P = 1.4e-14). **(b)** Ratio of the variance in trial-by-trial population activity explained by the 1st principal component in unadapted and adapted conditions for monkey R (red open circles, N = 8 sessions) and monkey T (blue open circles, N = 8 sessions). Each open circle represents the ratio evaluated for an individual session. Error bars (s.e.m) were evaluated by performing PCA analysis on sub-sampled trials in each condition. Light shaded circles represent sessions where the ratio was not significantly greater than 1.

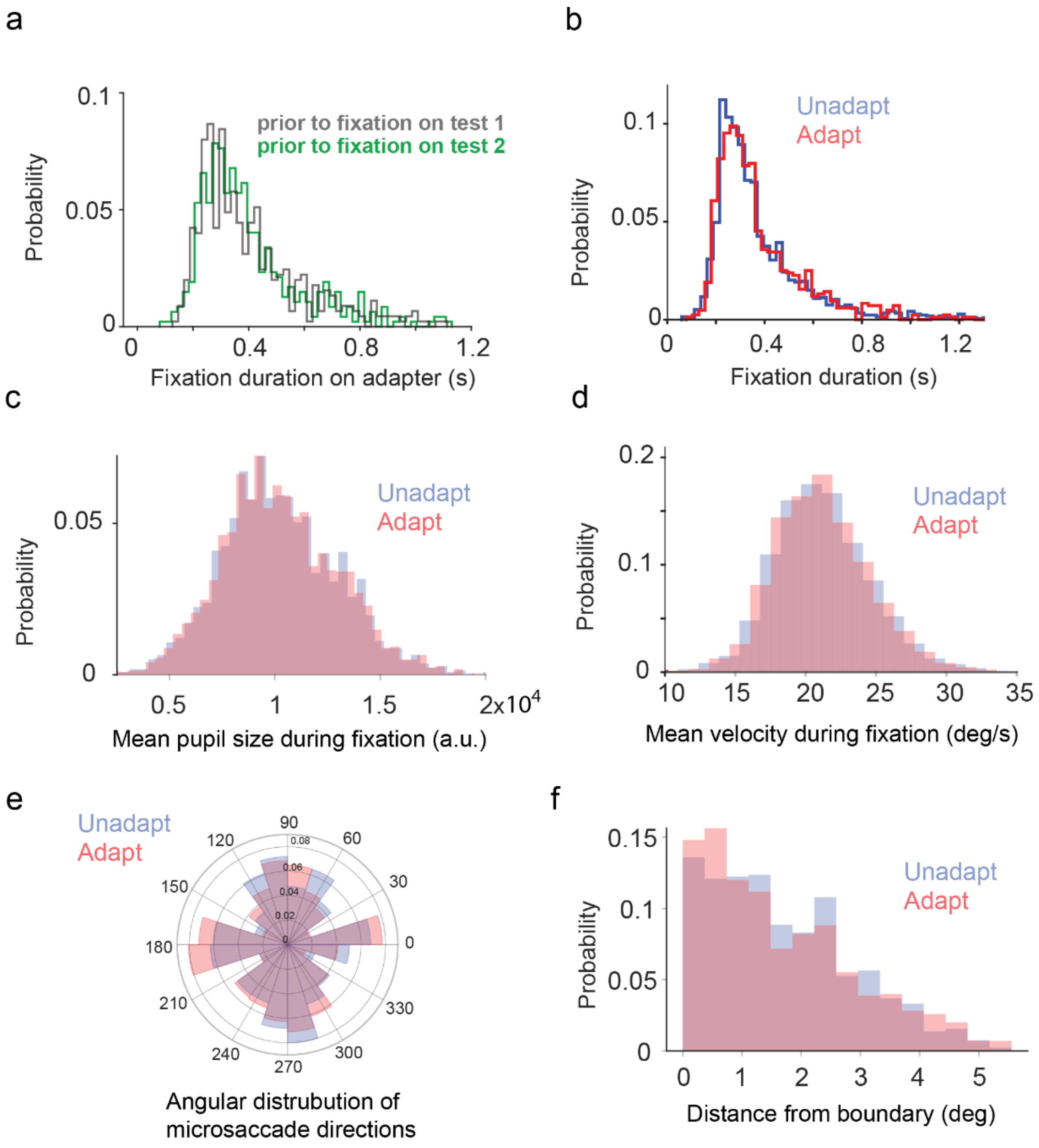

Supplementary Fig. 4 Fixation properties are not significantly different across conditions during free-viewing. (a) Distribution of fixation durations on the adapter prior to fixation on test stimuli 1 (gray histogram) and 2 (green histogram) ($P > 0.05$, two-sided Wilcoxon rank sum test). **(b)** Distribution of fixation durations (FD) on test stimuli for unadapted and adapted trials ($FD_{unad} = 380 \pm 5$ ms; $FD_{ad} = 385 \pm 7$ ms; $P > 0.05$, two-sided Wilcoxon rank sum test). **(c)** Distribution of mean pupil size (PS) during fixations on test stimuli for unadapted and adapted trials ($PS_{unad} = 1.03e4 \pm 64$ a.u; $PS_{ad} = 1.04e4 \pm 126$ a.u.; $P > 0.05$, two-sided Wilcoxon rank sum test). **(d)** Distribution of mean velocity (v) during fixations on test stimuli for unadapted and adapted trials ($v_{unad} = 21.3 \pm 0.1$ deg/s ms; $v_{ad} = 21.5 \pm 0.2$ deg/s; $P > 0.05$, two-sided Wilcoxon rank sum test). **(e)** Distribution of microsaccade directions detected during fixations on test stimuli in unadapted and adapted conditions **(f)** Distribution of mean distance (d) of edges of neurons receptive fields from stimulus boundaries during adapted and unadapted trials. ($d_{unad} = 1.69 \pm 0.02$ deg; $d_{ad} = 1.66 \pm 0.04$ deg; $P > 0.05$, Wilcoxon rank sum test).

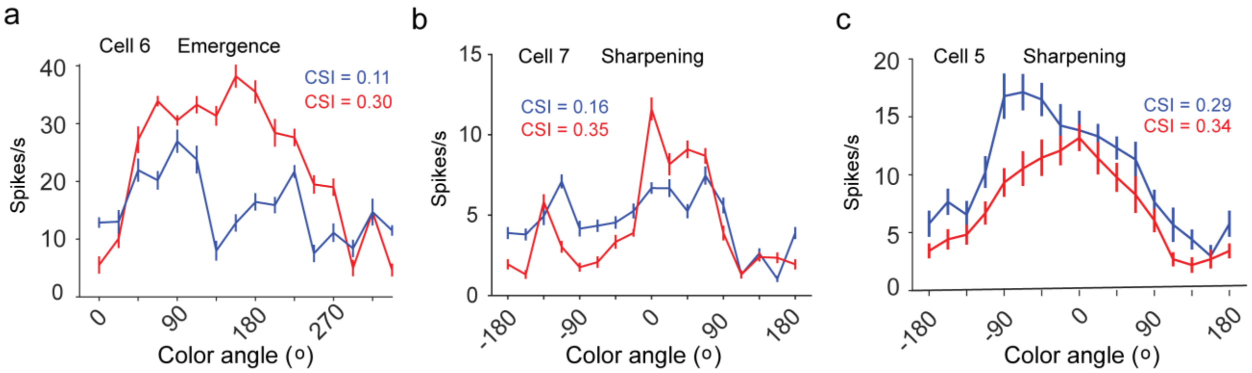

Supplementary Fig. 5 Emergence and sharpening of color tuning after adaptation to oriented gratings. (a) Example cell untuned to color in unadapted condition (blue) showing emergence of tuning to color ($CSI_{adapt} = 0.30$; Rayleigh's test, $P < 0.01$) after being adapted to a grating (red). **(b, c)** Two example cells exhibiting sharpening of existing tuning (Rayleigh's test, $P < 0.01$ for both unadapted and adapted conditions) post adaptation. Solid lines represent mean responses to color stimuli and errorbars represent s.e.m evaluated across multiple presentations of each color stimuli. Red curves represent adapted condition whereas blue curves represent tuning in unadapted condition.

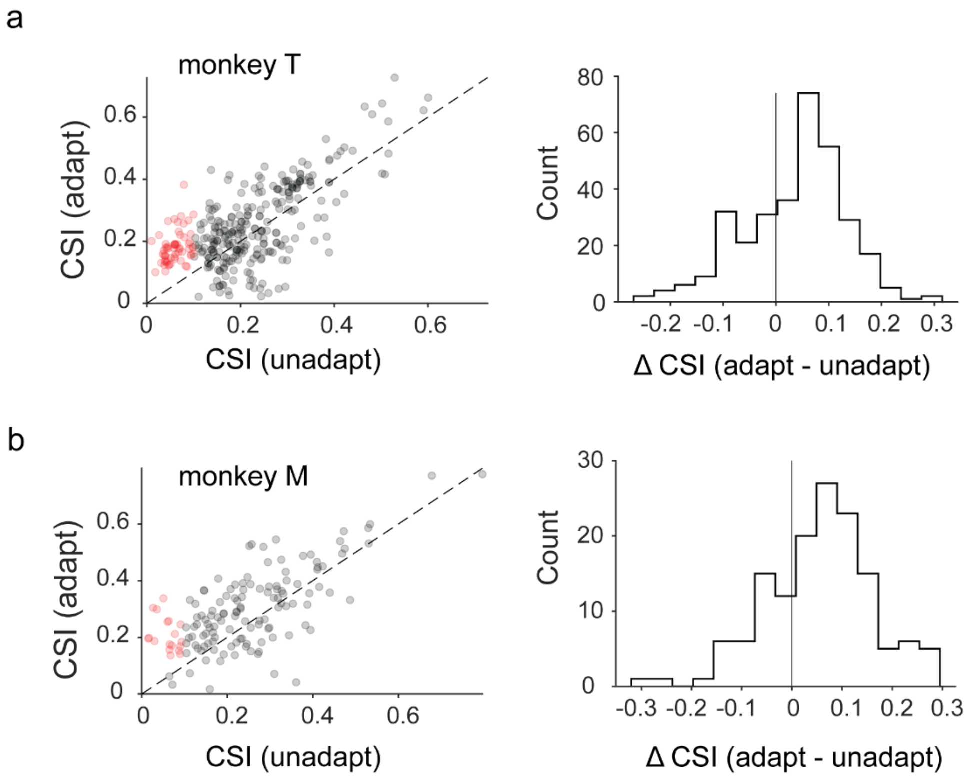

Supplementary Fig. 6 Cross feature adaptation sharpens color tuning in neural populations. (a) Left: Color selectivity index (CSI) for neurons in adapted and unadapted conditions (Monkey T, 263 neurons). Red circles represent neurons that were untuned to color in the unadapted condition but gained significant color tuning after orientation adaptation. Black circles represent all other neurons. **Right:** distribution of change in CSI values (adapt – unadapt, $\Delta CSI_{mean} = 0.04 \pm 0.003$, one-sided Wilcoxon signed rank test, P =3.3e-11) calculated for neurons across all sessions in monkey T. **(b)** Same as in a except for monkey M (123 neurons, mean $\Delta CSI = 0.05 \pm 0.001$, one-sided Wilcoxon signed rank test, P < 1.4e-8).

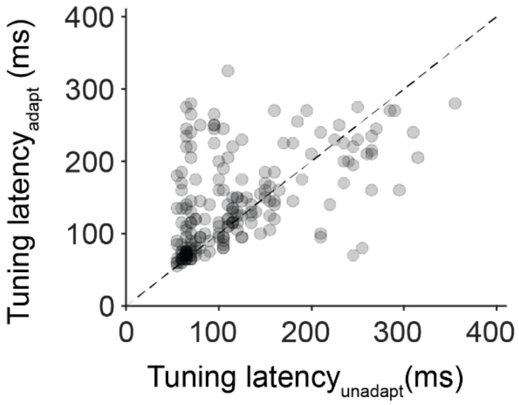

Supplementary Fig. 7 Cross-feature adaptation increases latency to peak tuning. Tuning latency (L) values (see Methods) for neurons significantly tuned under both conditions. An oriented grating was used as the adapter while tuning was examined for color stimuli. Each gray circle represents the tuning latency in both conditions for a single neuron. A significant increase in latency is observed post adaptation ($L_{unadapt}$ = 119 ± 5 ms, L_{adapt} = 140 ± 5 ms; two-sided Wilcoxon signed rank test, P = 1.2e-5).

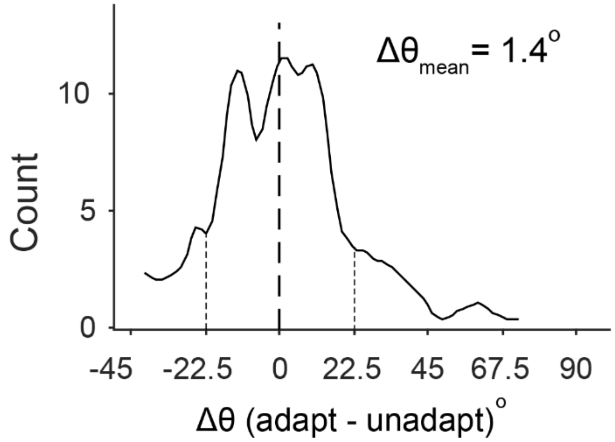

Supplementary Fig. 8 Modest changes in preferred color after cross-feature adaptation. Distribution of change in preferred color (Δθ) of neurons pooled across all sessions. Out of 343 neurons analyzed across both animals, only 18% showed changes in preferred color > 22.5°, i.e., the resolution of the color stimuli used in the experiments (16 equiluminant colors evenly distributed on the hue circle spanning 0 to 360°). Dotted vertical lines separate the distribution into regions greater or less than ±22.5° (resolution of the color stimuli in hue space, see Fig. 2a) and are included for visualization purposes.

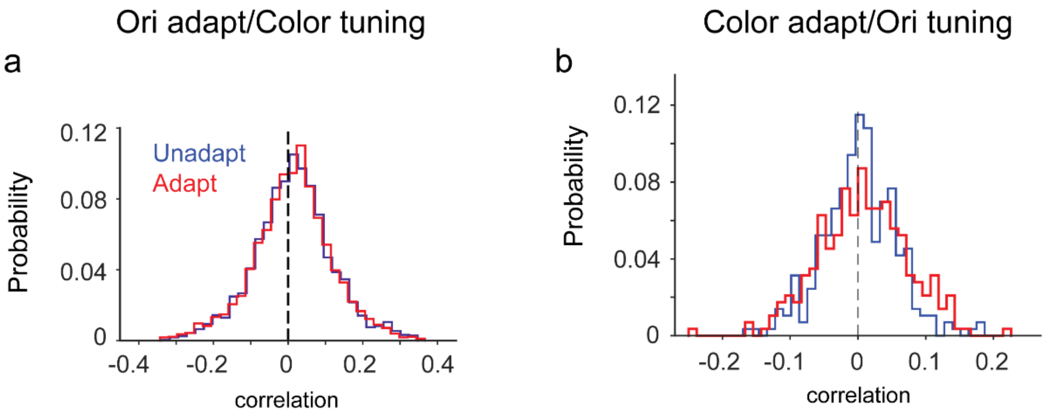

Supplementary Fig. 9 Distribution of pairwise correlations remain unchanged after cross feature adaptation (a) Distribution of pairwise noise correlations (n = 2817 pairs) in the unadapted (blue) and adapted condition (red) in the case of orientation adapt/color test. **(b)** Distribution of pairwise noise correlations (n = 288 pairs) before and after adaptation (color adapt/orientation test).

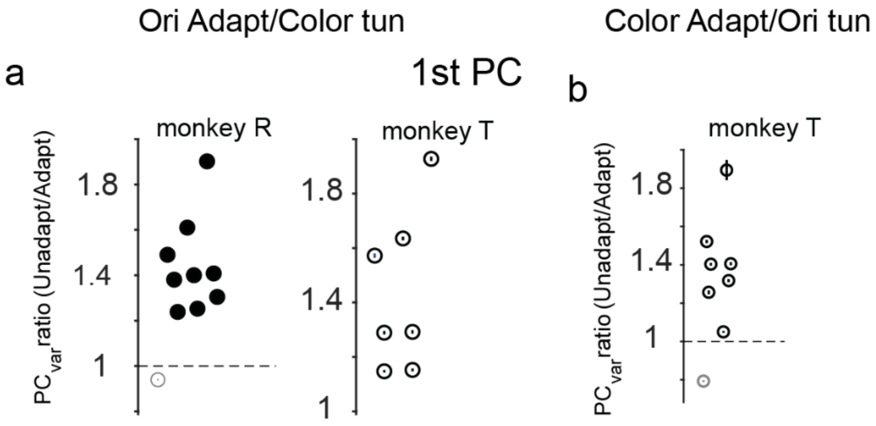

Supplementary Fig. 10 Higher variance in trial-by-trial population activity captured by the 1st principal component in unadapted versus adapted trials during passive fixation. (a) Mean values of the ratio of variance in trial-by-trial population activity explained by the 1st principal component in unadapted and adapted conditions (orientation adapter/color test) for each individual session in monkey R (solid black circles, N = 10 sessions; one-sided Wilcoxon signed rank test, P < 0.01 for filled black circles) and monkey T (open black circles, N = 7 sessions, ratio significantly > 1; one-sided Wilcoxon signed rank test, P < 0.01). Error bars (s.e.m) were calculated by performing PCA analysis multiple times (n = 100) on sub-sampled trials in each condition. Lighter shaded circles represent sessions where the ratio is not significantly greater than 1 (two-sided Wilcoxon signed rank test, P > 0.05). **(b)** Same as in a except for color adapter and orientation test stimuli (monkey T, N = 8 sessions).

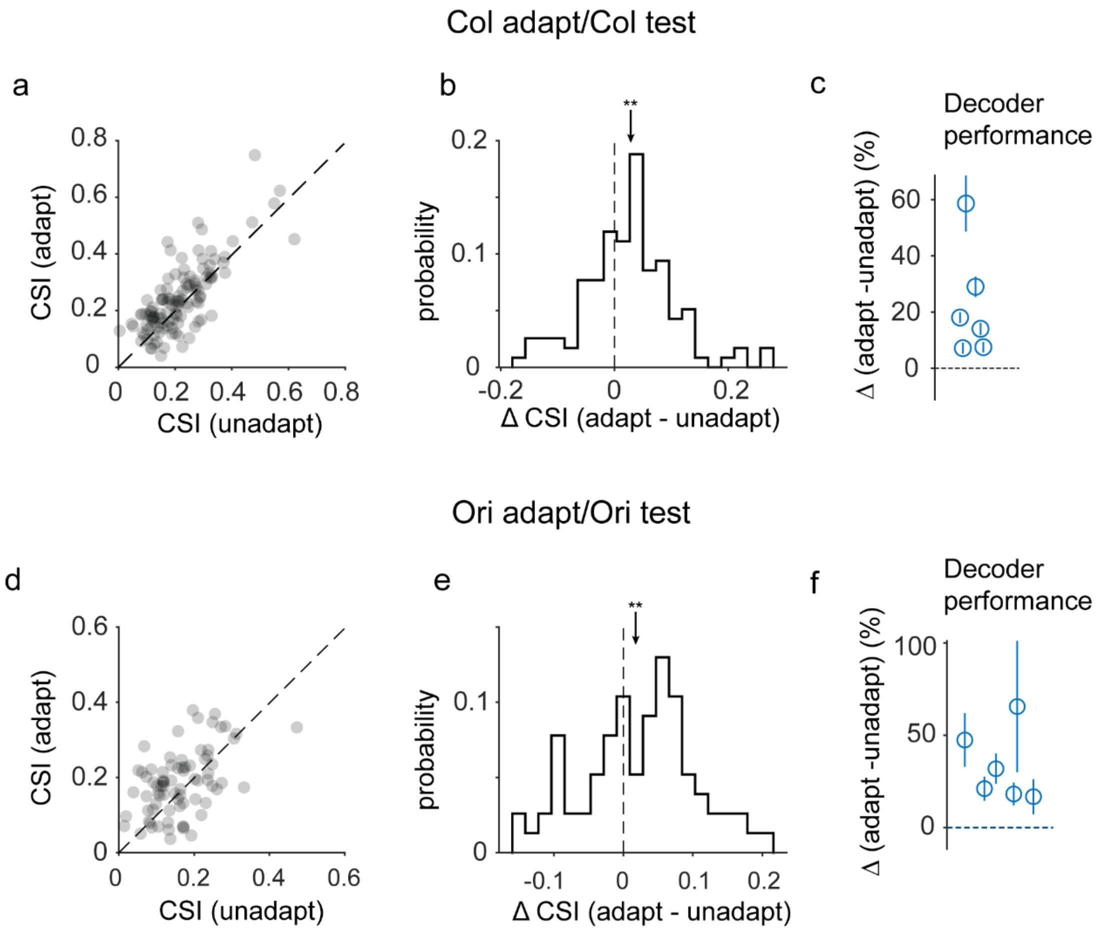

Supplementary Fig. 11 Iso-feature adaptation increases overall tuning strength and discriminability by de-correlating population responses to stimuli. (a) CSI values of color tuning in unadapt and adapt conditions for color adaptation (n = 114 neurons). **(b)** Histogram of difference in CSI values (adapt-unadapt). Arrow represents mean change in CSI and asterisks denote statistical significance of a two-sided Wilcoxon signed rank task (P = 3.7e-4). **(c)** Percentage change in decoder accuracy values (adapt - unadapt) for each session (N = 6 sessions). Each unfilled circle represents a session and error bars (s.e.m) were calculated by running the decoder multiple times (n = 500) with a different set of training and test trials. **(d-f)** Same as in a-c except for when the test and adapting stimulus were oriented gratings (n = 77 neurons, ΔCSI significantly > 0, two-sided Wilcoxon signed rank test, P = 0.01).
