# Supplementary material for: Adaptive coding across visual features during free-viewing and fixation conditions
Source: Nat Commun. 2023 Jan 6;14:87. doi: 10.1038/s41467-022-35656-w (PMC9816177; doi:10.1038/s41467-022-35656-w)
Supplement: Supplementary file 3 — Reporting Summary [file 41467_2022_35656_MOESM3_ESM.pdf]

nature portfolio

Corresponding author(s): Valentin Dragoi; Sunny Nigam

Last updated by author(s): Dec 2nd, 2022

Reporting Summary

Nature Portfolio wishes to improve the reproducibility of the work that we publish. This form provides structure for consistency and transparency in reporting. For further information on Nature Portfolio policies, see our Editorial Policies and the Editorial Policy Checklist.

Statistics

For all statistical analyses, confirm that the following items are present in the figure legend, table legend, main text, or Methods section.

n/a	Confirmed	
☐	☒	The exact sample size (*n*) for each experimental group/condition, given as a discrete number and unit of measurement
☒	☐	A statement on whether measurements were taken from distinct samples or whether the same sample was measured repeatedly
☐	☒	The statistical test(s) used AND whether they are one- or two-sided<br>*Only common tests should be described solely by name; describe more complex techniques in the Methods section.*
☒	☐	A description of all covariates tested
☐	☒	A description of any assumptions or corrections, such as tests of normality and adjustment for multiple comparisons
☐	☒	A full description of the statistical parameters including central tendency (e.g. means) or other basic estimates (e.g. regression coefficient) AND variation (e.g. standard deviation) or associated estimates of uncertainty (e.g. confidence intervals)
☐	☒	For null hypothesis testing, the test statistic (e.g. *F*, *t*, *r*) with confidence intervals, effect sizes, degrees of freedom and *P* value noted<br>*Give P values as exact values whenever suitable.*
☒	☐	For Bayesian analysis, information on the choice of priors and Markov chain Monte Carlo settings
☒	☐	For hierarchical and complex designs, identification of the appropriate level for tests and full reporting of outcomes
☐	☒	Estimates of effect sizes (e.g. Cohen's *d*, Pearson's *r*), indicating how they were calculated

Our web collection on statistics for biologists contains articles on many of the points above.

Software and code

Policy information about availability of computer code

Data collection	Data was collected using Utah arrays manufactured by Blackrock Neurotech
Data analysis	Data was analyzed using Offline Sorter v3.3.5 (Plexon Inc), readily available functions in MATLAB v.2019b (Mathworks) and functions in the CircStat Toolbox (v1.21.0.0).

For manuscripts utilizing custom algorithms or software that are central to the research but not yet described in published literature, software must be made available to editors and reviewers. We strongly encourage code deposition in a community repository (e.g. GitHub). See the Nature Portfolio guidelines for submitting code & software for further information.

Data

Policy information about availability of data

All manuscripts must include a data availability statement. This statement should provide the following information, where applicable:

- Accession codes, unique identifiers, or web links for publicly available datasets
- A description of any restrictions on data availability
- For clinical datasets or third party data, please ensure that the statement adheres to our policy

Source data for figure panels has been included in a zipped folder and presented as Supplementary Information. Raw data and code used in the study are available from the corresponding author upon request.

Human research participants

Policy information about studies involving human research participants and Sex and Gender in Research.

Reporting on sex and gender	N.A
Population characteristics	N.A
Recruitment	N.A
Ethics oversight	N.A

Note that full information on the approval of the study protocol must also be provided in the manuscript.

Field-specific reporting

Please select the one below that is the best fit for your research. If you are not sure, read the appropriate sections before making your selection.

☒ Life sciences ☐ Behavioural & social sciences ☐ Ecological, evolutionary & environmental sciences

For a reference copy of the document with all sections, see nature.com/documents/nr-reporting-summary-flat.pdf

Life sciences study design

All studies must disclose on these points even when the disclosure is negative.

Sample size	Sample sizes are cell counts and and the number of independent recordings performed on different days. The number of cells recorded cannot be pre-determined and are dependent on the signal quality of the chronically implanted array. Number of recorded sessions are consistent with those of multiple other studies in non-human primate electrophysiology.
Data exclusions	Visually unresponsive or low responsive cells, as well as unstable neurons were removed from the analysis.
Replication	Results were replicated across at least N = 8 sessions each for both free-viewing and passive fixation paradigms in 2 animals.
Randomization	All conditions were randomly interleaved.
Blinding	Blinding was not relevant as this study did not involve any clinical testing component

Reporting for specific materials, systems and methods

We require information from authors about some types of materials, experimental systems and methods used in many studies. Here, indicate whether each material, system or method listed is relevant to your study. If you are not sure if a list item applies to your research, read the appropriate section before selecting a response.

Materials & experimental systems

n/a	Involved in the study	
☒	☐	Antibodies
☒	☐	Eukaryotic cell lines
☒	☐	Palaeontology and archaeology
☐	☒	Animals and other organisms
☒	☐	Clinical data
☒	☐	Dual use research of concern

Methods

n/a	Involved in the study	
☒	☐	ChIP-seq
☒	☐	Flow cytometry
☒	☐	MRI-based neuroimaging

Animals and other research organisms

Policy information about studies involving animals; ARRIVE guidelines recommended for reporting animal research, and Sex and Gender in Research

Laboratory animals	Macaca mulatta, 3 males, monkey T 14 years old, Monkey R 11 years old, and Monkey M 10 years old.

Wild animals	Study did not involve wild animals
Reporting on sex	Study was performed on male macaques only
Field-collected samples	Study did not involve field-collected samples
Ethics oversight	All experiments were performed in accordance with protocols approved by the Animal Welfare Committee (AWC) and the Institutional Animal Care and Use Committee (IACUC) at the University of Texas Health Science Center at Houston (UTHealth)

Note that full information on the approval of the study protocol must also be provided in the manuscript.
